# Effect of Deoxynivalenol and Other Type B Trichothecenes on the Intestine: A Review 

**DOI:** 10.3390/toxins6051615

**Published:** 2014-05-21

**Authors:** Philippe Pinton, Isabelle P. Oswald

**Affiliations:** 1 INRA (Institut National de la Recherche Agronomique), UMR1331, Toxalim, Research Centre in Food Toxicology, Toulouse F-31027, France; E-Mail: Philippe.Pinton @toulouse.inra.fr; 2 Université de Toulouse, Institut National Polytechnique, UMR1331, Toxalim, Toulouse F-31000, France

**Keywords:** barrier function, food-contaminant, immune response, intestinal lesions, mycotoxins

## Abstract

The natural food contaminants, mycotoxins, are regarded as an important risk factor for human and animal health, as up to 25% of the world’s crop production may be contaminated. The *Fusarium* genus produces large quantities of fusariotoxins, among which the trichothecenes are considered as a ubiquitous problem worldwide. The gastrointestinal tract is the first physiological barrier against food contaminants, as well as the first target for these toxicants. An increasing number of studies suggest that intestinal epithelial cells are targets for deoxynivalenol (DON) and other Type B trichothecenes (TCTB). In humans, various adverse digestive symptoms are observed on acute exposure, and in animals, these toxins induce pathological lesions, including necrosis of the intestinal epithelium. They affect the integrity of the intestinal epithelium through alterations in cell morphology and differentiation and in the barrier function. Moreover, DON and TCTB modulate the activity of intestinal epithelium in its role in immune responsiveness. TCTB affect cytokine production by intestinal or immune cells and are supposed to interfere with the cross-talk between epithelial cells and other intestinal immune cells. This review summarizes our current knowledge of the effects of DON and other TCTB on the intestine.

## 1. Introduction

Mycotoxins are structurally diverse fungal metabolites that can contaminate a variety of dietary components consumed by animals and humans. It is estimated that 25% of the world’s crop production is contaminated by mycotoxins during the pre-harvest period, transport, processing or storage [1]. The major mycotoxin-producing fungal genera are *Aspergillus*, *Fusarium* and *Penicillium*, mainly producing aflatoxins, zearalenone, trichothecenes, fumonisins, ochratoxins and ergot alkaloids.

**Figure 1:**
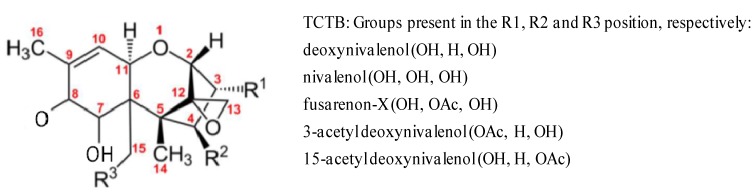
Chemical structure of Type B trichothecenes. TCTB, Type B trichothecenes.

Among the mycotoxins produced by the *Fusarium* genus, the broad family of trichothecenes (TCT) is extremely prevalent. They represent the most diverse chemical group of all the mycotoxins, and their molecular weights range between 200 and 500 Da. All TCT possess a sesquiterpenoid structure with or without a macrocyclic ester or an ester-ether bridge between C-4 and C-15. They contain a common 12,13-epoxytrichothecene group responsible for their cytotoxicity and a 9,10-double bond with various side chain substitutions. The non-macrocyclic TCT constitute two groups: Type A, including T-2 toxin, HT-2 toxin, neosolaniol and diacetoxyscirpenol (DAS), while the Type B group contains a ketone and includes fusarenon-X (FUS-X), nivalenol (NIV) and deoxynivalenol (DON) and its 3-acetyl and 15-acetyl derivatives (3- and 15-ADON) (Figure 1). The number and position of the hydroxyl and acetyl-ester groups can influence the relative toxicity within eukaryotic cells. Their relative capacity to interfere with protein synthesis has been attributed to a combination of different factors: the rate of transport into cells, metabolism by cytosol enzymes, changes in affinity for the active binding site or the ability to interfere with protein synthesis [2].

Deoxynivalenol and other Type B TCT (TCTB) are commonly found in cereals, such as wheat, rye, barley, oats and corn, all over the world [2,3,4]. These toxins are resistant to milling, processing and heating and, therefore, readily enter the food chain [5]. The total intake of DON in microgram per kilogram of body weight per day has been estimated to reach from 0.78 in an African diet to 2.4 in a Middle Eastern diet [6]. Intoxications following the consumption of foodstuffs contaminated with TCT have occurred in both humans and animals, with large numbers of people and livestock being affected [4]. Many outbreaks of acute human disease involving nausea, vomiting, gastro-intestinal upset, dizziness, diarrhea and headache have been reported in Asia [7,8]. These outbreaks have been attributed to the consumption of *Fusarium*-contaminated grains and, more recently, to the presence of DON at reported concentrations of 3–93 mg/kg in grain for human consumption [6].

More than 40 countries have introduced regulatory or guideline levels for DON in food and feed. In the USA, the Food and Drug Administration (FDA) has established an advisory level of 1 ppm of DON for bran, flour and germ targeted for human consumption [9]. The European Commission decided to limit the level of DON in food from 0.2 to 1.75 mg/kg for cereals and derived products, depending on the exposed population, and in feed from 0.9 to 5 mg/kg for complementary and complete feedstuffs, depending on the species [10,11].

Following the ingestion of contaminated food or feed, intestinal epithelial cells may be exposed to high concentrations of toxicants, potentially affecting intestinal functions [12]. The intestinal epithelium is a single layer of cells lining the gut lumen that acts as a selective filter, allowing the translocation of essential dietary nutrients, electrolytes and water from the intestinal lumen into the circulation. It also constitutes the largest and most important barrier to prevent the passage from the external environment into the organism of harmful intraluminal substances, including foreign antigens, microorganisms and their toxins [13]. The establishment of the epithelial monolayer by intestinal epithelial cells is dependent upon a considerably high degree of intracellular and intercellular organization. Within each epithelial cell, structural integrity is maintained by the presence of a complex cytoskeletal network of microfilaments playing a crucial role in maintaining cellular polarity and in supporting points of cell-cell contact. The interaction and contact between adjacent intestinal epithelial cells of the monolayer is mediated by distinct junctions, including tight junctions, desmosomes and adherens junctions [14]. The function as a selective permeable barrier places the mucosal epithelium at the center of interactions between the mucosal immune system and luminal contents, which includes dietary antigens and microbial products [15]. The intestine is a privileged immune site, where immunoregulatory mechanisms simultaneously defend the body against pathogens, but also preserve tissue homeostasis to avoid immune-mediated pathology in response to environmental challenges.

This review summarizes the consequences of exposure to DON and other TCTB on histopathological intestinal lesions, on the potential disruption of the intestinal barrier function and on the active role of the intestinal mucosa in immune responsiveness. We will focus on the data obtained in humans, laboratory animals, poultry and pigs, as this latter species can be regarded as a good model for man [16,17].

## 2. DON and Other TCTB Reduce Growth

The reduction in weight gain as a consequence of reduced feed consumption is strongly associated with the exposure of farm animals to DON, with pigs being one of the most sensitive species. Current regulatory standards for DON in foods are based on its ability to cause growth suppression [18]. While DON is considered one of the least lethal TCT, its anorexic and emetic potencies are equal to, or greater than, those reported from the more acutely toxic TCT, such as T2-toxin [19]. Recently, the anorectic potencies of TCTB were compared in mice, following intraperitoneal or oral exposure: NIV and FUS-X were shown to have greater effects than DON, which had similar effects as 3- and 15-ADON [20]. The ability of DON to induce anorexia may be the consequence of the dysregulation of various signaling pathways. DON may act at different levels to induce impaired growth and weight gain, including on neuroendocrine signaling, immune responses, growth hormone or a central neuronal network. The involvement of neuroendocrine factors, such as serotonin, has been proposed [21,22]. Serotonin is produced and released by the enterochromaffin cells in the gut, acts as a paracrine on the enteric nervous system [23] and can affect the secretion of both anorexigenic or orexigenic hormones [24]. The impact of DON on immune responses can also affect feed consumption, because the activation of proinflammatory cytokines is recognized as a cause of anorexia [24]. Indeed, these toxins induce several suppressors of cytokine signaling (SOCS) [25] that impair growth hormone signaling by suppressing two growth-related proteins, the hepatic insulin-like growth factor acid-labile subunit (IGFALS) and insulin-like growth factor 1 (IGF1), as demonstrated in mice [25]. In this species, DON also induces the release of the satiety hormones, peptide YY (PYY) and cholecystokinin (CCK), proposed as critical mediators of DON-induced anorexia [26]. The impact of DON exposure on the central regulation of energy balance has been studied in mice and pigs [27,28,29]. Recently, Girardet *et al*. [29] showed that in addition to its peripheral action, DON can reach the brain after *per os* administration and act centrally on the anorexigenic/orexigenic balance.

In conclusion, most data describing the effects of DON on food intake were obtained in mice or in pig, and they point out both central and peripheral neuroendocrine control mechanisms. Neuroendocrine factors and proinflammatory cytokines drive the anorexigenic effect of DON. Recent experiments obtained only in rodent demonstrated that anorexia is induced rapidly within a few minutes following DON ingestion. Complementary studies are needed to evaluate if the mechanisms involved in anorexia are similar in rodents and other species.

## 3. DON and Other TCTB Affect Nutrient Absorption

The intestinal epithelium mediates the selective permeability from the intestinal lumen into the circulation of essential dietary nutrients, electrolytes and water through two major routes: transcellular permeability, generally associated with solute transport through the epithelial cells and predominantly regulated by selective transporters for amino acids, electrolytes, short-chain fatty acids and sugars; paracellular permeability, associated with transport via the space between epithelial cells and regulated by intercellular membrane junctional complexes [13]. The intestinal epithelium is a recognized target for NIV and FUS-X with acute effects, such as impaired sugar and electrolyte absorption [30]. The impaired absorption of nutrients may participate in the effect of TCT on animal growth [31]. The impacts of DON and other TCTB on nutrient absorption or transport at the intestinal level are summarized in Table 1.

### 3.1. Humans

In the human intestinal epithelial cell line, HT-29-clone D4, exposure to 10 µM of DON affects the activities of intestinal transporters: inhibition of the D-glucose/D-galactose sodium-dependent transporter (SGLT1), of the D-fructose transporter, glucose transporter-5 (GLUT5), and of active and passive L-serine transporters has been observed. The transport of palmitate was increased, whereas the uptake of cholesterol was not affected by the mycotoxin. At high concentrations (100 µM), SGLT1 activity was inhibited, whereas the activities of all the other transporters were increased [32].

### 3.2. Rodents

Exposure of mice to DON at 10 mg/kg for six weeks did not modulate the absorption of water, L-leucine, L-tryptophan, iron or D-glucose. However, a slight, but significantly reduced, transfer of glucose was observed. Furthermore, a significant decrease (up to 50%) in the transfer, as well as tissue accumulation of 5-methyltetrahydrofolic acid in the jejunal segment was observed. These findings indicate that subchronic ingestion of DON can impair the intestinal transfer and uptake of nutrients [33].

**Table 1:** Effect of TCTB exposure on nutrient absorption. DON, deoxynivalenol; NIV, nivalenol; 15-ADON, 15-acetyl derivative of DON; FUS-X, fusarenon-X.

Toxin	Animal species	Concentration and duration of exposure	Effects on nutrients absorption	References
DON	Human HT-29 cell line (*in vitro*)	10 µM 48 h	Inhibition of D glucose/D galactose transporters	[32]
			Inhibition of D-fructose transporter Inhibition of the active L-serine transporter	
			Inhibition of active and passive L-serine transport	
			Increase in palmitate transport	
DON	Mouse (*in vivo*)	10 mg/kg feed 6- weeks	Reduced weight gain	[33]
			Decreased transfer of glucose	
			Decreased jejunal transfer and tissue accumulation of 5-methyltetrahydro folic acid	
	Poultry (*ex vivo*)	33 µM 30 min	Inhibition of jejunal Na^+^-amino acid co-transport	[34]
	Poultry (*ex vivo*)	33 µM 30 and 45 min	Decrease in jejunal glucose uptake	[35]
NIV	Poultry (*ex vivo*)	33 µM 30 min	Decrease in jejunal glucose uptake	[31]
15-ADON	Poultry (*ex vivo*)	33 µM 30 min	Decrease in jejunal glucose uptake	[31]
FUS-X	Poultry (*ex vivo*)	33 µM 30 min	No obvious effect	[31]

### 3.3. Farm Animals

Several studies have investigated the effects of DON on farm animals, with most of the data obtained in chickens. The electrophysiological properties of chicken intestinal mucosa exposed to DON were evaluated using isolated jejunum fragments in Ussing chambers [34]. Intestinal transport was determined by changes in the short-circuit current (Isc), as a measure of ion transmembrane flux, in the middle segment of the jejunum of broilers. The addition of D-glucose produced an increase in the Isc, and this was reversed by different TCTB, including DON [31].

The Isc was decreased by the addition of L-proline on the luminal side of the isolated mucosa after DON treatment, an effect that could be attributed to a strong inhibition of the L-proline/sodium-dependent transporter by DON [34].

Cotransporters are specialized membrane proteins using electrochemical gradients across the membrane for transporting sugars, amino acids and ions. The inhibition of Na^+^ transport and Na^+^-D-glucose co-transport are important mechanisms of DON toxicity in the intestine of chickens [34,36,37]. Indeed, DON treatment (33.7 µM) decreased glucose uptake almost as efficiently as phlorizin, a specific inhibitor of the sodium-dependent glucose cotransporter, SGLT-1 [35]. 

When comparing the different TCTB, the activity of the glucose co-transporter appears to be more sensitive to DON, NIV and 15-ADON than to FUS-X in the jejunum of broilers [31]. 

In Ross broilers fed either a basal, low DON or high DON diet (0.26, 1.68 and 12.21 mg/kg of DON, respectively), a progressive decrease in the relative density (weight:length) of the small intestine with increasing time of exposure was observed, which could be correlated with a decrease in villus height in the small intestine. The Isc of the jejunal epithelium was reduced in birds fed the high DON diet [38]. Recently, morphometric analysis of duodenal sections of hybrid turkey poults, fed for three weeks with DON at 5.2 mg/kg demonstrated a significant reduction in villus height and apparent villus surface area [39]. Using a global transcriptomic approach, Dietrich *et al*. [40] identified, in the jejunum of broilers fed for 23 days with DON at 2.5 or 5 mg/kg, a downregulation of genes involved in the nutrient uptake into jejunal cells: *SLC2A5*, which facilitates glucose and fructose transport, *SLC27A4*, involved in the palmitate transport and *SLC16A1*, involved in monocarboxylate uptake.

In conclusion, the data obtained by *in vitro* and *ex vivo* experiments performed in different species (human, mouse and poultry) show that DON affects the absorption of amino acids and sugars in intestinal epithelial cells. These studies indicate that DON affects key nutrients transporters. The precise mechanisms of action of DON and other TCT is still unknown, but these toxicants could act on the transporter proteins themselves and also on other constituents of the “transportsome”, such as regulatory molecules or scaffold proteins.

## 4. DON and Other TCTB Induce Intestinal Lesions

Chronic exposure to TCTB induces digestive problems, reduced food intake and food refusal. The most striking clinical sign is the alteration of growth performance, most often related to decreased feed intake and decreased weight gain (Table 2). The reporting of intestinal lesions has been inconsistent and not systematically correlated with the clinical signs. Animal species differ in their susceptibility to these toxins. For example, as far as DON is concerned, the animal species can be ranked in the following order: pigs > mice > rats > poultry ≈ ruminants [2].

Among different hypotheses, the interaction of mycotoxin-microbiota can be proposed to explain, at least in part, the differences between species. The microbiota is thought to play important roles in the maturation of the intestinal and immune systems, in the nutrition of the host and, finally, in its protection against pathogenic micro-organisms and hazardous chemicals/xenobiotics, including TCTB. Differences in the localization of the gut bacteria able to convert toxic DON into its non-toxic de-epoxide metabolite, DOM-1, prior to or after the small intestine can have a major effect on the bioavailability of ingested TCTB. On this basis, animals can be divided into two groups: polygastric animals and birds with a high bacterial content located both before and after the small intestine; and monogastric species (including humans, pigs and rodents) with a high bacterial content located only after the small intestine, *i.e.*, in their colon [41].

**Table 2:** Intestinal lesions reported after TCTB exposure.

Toxin	Animal species	Concentration and duration of exposure	Intestinal Lesions	References
**Repeated exposure to TCTB (dietary or gavage)**				
**DON**	**Pig**	0.75–4.2 mg/kg 3–5 weeks	Edema and congestion	[42,43,44]
		0.7–5.8 mg/kg 4 weeks	Slight to moderate inflammation and congestion of intestinal mucosa. Slight to moderate degeneration of lymphoid cells in Peyer’s patches and in lymph nodes	[45]
		4 mg/kg	Corrugations in the fundic region (stomach)	[46]
		2–3 mg/kg 4 weeks	Corrugations in jejunum	[47]
		2.8 mg/kg	Multifocal atrophy and villus fusion, Apical necrosis of villi,	[48,49]
		5 weeks	Cytoplasmatic vacuolation of enterocytes, Edema of lamina propria Decrease in villus height Decrease in the number of goblet cells in the jejunum and the ileum	
	**Rat**	10 mg/kg 4 weeks	Alteration in villus architecture of the jejunum (increased villus fusion and shorter villus length). Increased apoptosis score in jejunal epithelial cells in association with higher number of mitotic cells and crypt fission	[50]
**DON 15-ADON**	**Pig**	DON 2.3 mg/kg	Reduction in villus height greater in presence of DON + 15-ADON compared to DON	[51]
		DON 1.2 mg/kg+ 15-ADON 0.9 mg/kg 4 weeks	Histological scores of the jejunum lower in animals fed DON + 15-ADON compared to DON	
***Ex vivo* short-term exposure to TCTB**				
**DON** **3-ADON** **15-ADON**	**Pig explants**	10 µM 4 h	Flattened and coalescent villi Lyses of enterocytes Interstitial edema and apoptosis 15-ADON >> DON = 3-ADON	[51]

The gut is identified as one of the target organs for TCTB, but no particular intestinal segment appears more sensitive than others [52,53]. The difference in sensitivity between species may be explained by differences in absorption, distribution, metabolism and elimination of DON [2]. The exact mechanism of the cellular entry of TCTB is not well characterized, and one can speculate that differences exist between species, leading to a differential sensitivity to these compounds. Moreover, the biotransformation of TCT by the detoxifying enzymes in the liver is highly variable between species. In the rat or pig liver, there is no de-epoxidation of DON, while these species are able to carry out glucurono-conjugation.

### 4.1. Humans

Long-term exposure to *Fusarium* toxins has been associated with an increased incidence of esophageal cancer in China. DON and also Fumonisin B1 were suspected to be a risk factor [54,55], though the role of these toxins needs to be clarified [2].

### 4.2. Rodents

In mice, after two years of exposure to NIV at a 30 mg/kg dietary concentration, the survival rate was generally higher in the NIV- treated animals than in the controls, where naturally occurring tumors, mostly lymphomas, were observed. No intestinal lesions were observed after two years of NIV exposure [56]. 

In weaning rats, after 28 days of exposure to DON-contaminated feed at 10 mg/kg, a decreased feed intake was observed, associated with a reduced weight gain. Lesions were observed in the jejunum: the villus architecture was altered, with an increase in villus fusion and a shorter villus length. The apoptotic score was increased in jejunal epithelial cells and associated with a greater number of mitotic cells and crypt fission [50].

### 4.3. Pigs

Congestion and erosions of the gastric and intestinal mucosae have been described following chronic DON exposure in pigs [42,43,44,45]. At 4 mg/kg of diet, DON may cause corrugations in the fundic region of the stomach [46], which were also observed at the jejunal level with lower doses of toxin (2–3 mg/kg diet) [47]. After five weeks of exposure to DON at 2.8 mg/kg of the diet, in the absence of changes in the pigs’ body weight, significant histological changes were observed. Indeed, multifocal atrophy and villus fusion, apical necrosis of villi, cytoplasmatic vacuolation of enterocytes and edema of lamina propria were detected in the jejunum and ileum of DON-treated pigs [48]. A significant decrease in villus height was observed in the jejunum, probably reflecting a change in the balance between epithelial cell proliferation and apoptosis [49], while no difference in crypt depth was observed in any intestinal region. The number of goblet cells that synthesize and secrete mucin, involved in gut barrier function, decreased significantly in the jejunum and the ileum of piglets fed DON [49]. Similarly, a significant reduction in villus height and in the number of goblet cells was observed in pig jejunal explants exposed to 10 µM DON for four hours [57]. When comparing the effects of DON and its acetylated derivatives, Pinton *et al*. [51] observed that the reduction in villus height was greater in animals receiving DON + 15-ADON than in the animals receiving feed contaminated only with DON. The histological scores of the jejunum reflecting the main histological changes were lower in animals fed with DON + 15-ADON compared with animals fed DON.

To conclude, comparable macroscopic lesions of the intestinal epithelium are observed in rodents and pig exposed to TCTB. The exact mechanisms leading to these lesions are not characterized; it is especially important to delineate the effect of DON on: (i) the alteration of intestinal epithelial cells; (ii) the repair of these cells; and (iii) the coalescence and shortening of villi. Future research should focus on the differential effects of TCT on the potential target cells in the intestinal epithelium (crypts and stem cells *vs.* villus and differentiated cells). The effect of TCT on the factors that control the balance between cell proliferation, differentiation and cell death should also be analyzed.

## 5. DON and Other TCTB Alter Intestinal Barrier Function

The toxicity of DON and other TCTB is partially explained by the ability of these compounds to bind to eukaryotic ribosomes [58] and to rapidly activate the mitogen-activated protein kinases (MAPKs) via a process termed the “ribotoxic stress response”. The MAPK cascades are central signaling pathways that regulate a wide variety of stimulated cellular processes, including proliferation, differentiation, apoptosis and stress response [59]. 

At present, the four different MAPK cascades identified are named according to their MAPK components: extracellular signal-regulated kinase 1 and 2 (ERK1/2), c-Jun N-terminal kinase (JNK), p38 and ERK5. Two possible upstream signal transducers for the DON-induced MAPK activation are the double-stranded RNA-activated protein kinase (PKR) and the hematopoietic cell kinase (Hck), a Src-family tyrosine kinase [24]. The consequence of this activation is an increase in proinflammatory gene expression, and the downstream effects include anorexia, reduced weight gain, immune stimulation, tissue injury and apoptosis. DON modulates cytokine and chemokine gene expression [60]. Highly dividing cells, such as intestinal epithelial cells or immune cells, are especially sensitive to TCTB, and the exposure of intestinal epithelial cells to these toxins may alter their ability to proliferate and to ensure a proper barrier function.

### 5.1. Effects on Cell Proliferation and Differentiation

In order to maintain an effective barrier function, the intestinal epithelium rapidly regenerates entirely in approximately one week, throughout life. Mature cells derived from intestinal stem cells migrate upwards along the crypt-villus axis towards the tip of the villus, gradually differentiating as they come closer to the tip [61]. Several studies have investigated the effects of mycotoxins on intestinal epithelial cell proliferation and on intestinal morphology (Table 3).

#### 5.1.1. Effects on Cell Growth

The effect of TCT on intestinal epithelial cell growth has mainly been studied in the two human cell lines, Caco-2 and HT-29 [32,62,63]. When treated with a range of concentrations of DON (84 nM to 84 µM), Caco-2 cells showed a reduction in protein synthesis, proliferation and survival [64]. Dividing Caco-2 cells were found to be more sensitive compared to differentiated cells [65]. The greater sensitivity of proliferating cells is probably due to the capacity of the toxin to inhibit protein synthesis and, subsequently, nucleic acid synthesis [66]. DON was also demonstrated to decrease the cell proliferation in the porcine intestinal epithelial cell line, IPEC-1, whereas the acetylated derivatives exhibited differential effects. Indeed, 3-ADON was less toxic and 15-ADON was equally toxic as DON [51]. In the IPEC-J2 porcine intestinal epithelial cell line, the cytotoxicity of DON was correlated with an increase in lactate dehydrogenase release and decrease in ATP content [67]. In the human intestinal cell line HT-29, the cytotoxic effect of DON was not correlated with the induction of the heat shock protein, Hsp 70, or with the generation of reactive oxygen species, but was associated with a fragmentation of DNA and the activation of the apoptotic molecules, p53 and caspase-3 [68]. In intestinal cells from rat species (IEC-6 cell line), DON at 10 µM reduced the viability and induced apoptosis, independently of any cell cycle arrest, but involving caspase-3 activation [69].

**Table 3:** Effect of TCTB exposure on intestinal barrier function.

Toxin	Animal species	Concentration and duration of exposure	Effects on barrier function	References
***In vivo* approach**				
**DON**	**Mouse**	acute exposure 25 mg/kg bw (one gavage)	Increase in 4 kDa dextran permeability Effect on the distribution pattern of claudin 1, 3 and 3 tight junction proteins in small intestine	[70]
	**Rat**	chronic exposure 2 mg/kg feed 28 days	Decrease in transepithelial electrical resistance (TEER) Increase in 4 kDa dextran permeability	[50]
***In vitro* approach: intestinal epithelial cell lines**				
**DON**	**Human HT-29** **cell line**	2 to 50 µM 24 h 10 µM 0–24 h	Dose dependent inhibition of cell viability (IC_50 _= 10 µmol/L) Time dependent: Increase in total DNA damage Increase in p53 protein level Increase in caspase-3 activity	[68]
	**Human Caco-2** **cell line**	84 µM 24 h	Decreased survival rate of 40%	[64]
	**Human HT-29** **cell line**	0.13 to 0.7 µM 6 to 15 d	Decrease in brush border enzyme activity Decrease in protein content Decrease in transepithelial electrical resistance (TEER) Increase in lucifer yellow permeability	[62]
	**Human Caco-2** **cell line**	30 µM 48 h	Decrease in TEER Decrease in claudin-4 tight junction proteins Increase in 4 kDa dextran permeability	[71]
	**Human Caco-2** **cell line**	10 µM 12 h	Increase in *E coli* K12 translocation	[72]
	**Human Caco-2** **cell line**	1.7 to 17 µM 24 h	Decrease in claudin-4 tight junction proteins	[73]
**DON**	**Porcine IPEC-1** **cell line**	10 to 50 µM 48 h	Decrease in TEER Decrease in claudin-3 and 4 tight junction proteins Increase in 4 kDa dextran permeability Increase in *E coli* 28C translocation	[71]
**DON** **3-ADON** **15-ADON**	**Porcine IPEC-1** **cell line**	10 to 30 µM	Decrease in TEER and increase in 4 kDa dextran permeability 15-ADON >> DON > 3-ADON	[51]
		24 to 48 h	Decrease in claudin-3 and -4 tight junction proteins expression 15-ADON >> DON = 3-ADON	
**DON**	**Porcine IPEC-J2** **cell line**	2.5 to 10 µM 24 h	Decrease in cell viability Increase of lactate dehydrogenase release Decrease in ATP content	[67]
***Ex vivo* approaches**										
**DON**	**Porcine tissue (Ussing chamber)**	20 to 50 µM 2 h	Increase in 4 kDa dextran permeability	[71]
	**Porcine tissue (jejunal explants)**	1 to 10 µM 4 h	Shortened and coalescent villi, lysis of enterocytes, edema	[48]

As the intestine is potentially exposed to mixtures of mycotoxins [74,75], Alassane-Kpembi *et al*. evaluated interactions caused by co-exposure to TCTB on proliferating Caco-2 cells [76]. Using the MTT test and neutral red uptake, the authors observed that binary or ternary mixtures show synergistic effects when toxins were at low concentrations (cytotoxic effect between 10% and 40%) and additive or nearly additive effects at higher concentrations (cytotoxic effect around 50%).

#### 5.1.2. Effects on Cell Differentiation

Using scanning electron microscopy, Kasuga *et al*. [62] demonstrated on differentiated Caco-2 cells that the formation of the brush border and the expression of two membrane-associated hydrolases related to enterocyte differentiation were affected by DON in a dose-dependent manner [62].

In broiler chicks, the ingestion of DON-contaminated feed produced an alteration in the small intestinal morphology, especially in the duodenum and jejunum, where the villi were shorter and thinner [77].

### 5.2. Effects on Barrier Functions

Polarized cells form strong barriers through the development of tight junctions between them. The intercellular tight junction is the rate-limiting barrier in the paracellular pathway for permeation by ions and larger solutes [78]. The investigations concerning the effects of TCTB on the intestinal barrier functions are only just beginning.

#### 5.2.1. Effects on TEER

The transepithelial electrical resistance (TEER) of cell monolayers can be considered a good indicator of the degree of organization of the tight junctions within the cell monolayer and epithelial integrity [79]. Several studies have investigated the effect of TCTB on the TEER of intestinal epithelial cell lines (Table 3). In three different human intestinal epithelial cell lines, HT-29, Caco-2 and T84, DON was found to induce a dose-dependent decrease in the TEER [32,62,63,71,72,80]. The same effect was observed in the porcine intestinal epithelial cell lines IPEC-1 and IPEC-J2 [71,81]. Interestingly, IPEC-1 cells showed greater sensitivity to DON compared with Caco-2 [71]. Several hypotheses can explain this higher sensitivity of IPEC-1 cells [71]. Firstly, Caco-2 cells were obtained from an adenocarcinoma, whereas IPEC-1 cells were derived from normal newborn piglets [82]. Secondly, even if Caco-2 cells express many morphological and biochemical characteristics of small intestine [83], they are derived from the colon. By contrast, IPEC-1 cells were obtained from jejunum and ileum. Thirdly, these two cell lines are from different species (pig *versus* human), and among animal species, pig is the species most sensitive to DON. However, it is difficult to assess the susceptibility of humans [2]. Interestingly, a differential sensitivity to DON of the TEER of intestinal cells from the IPEC-J2 cell line has been observed depending on the route of application. Indeed, following basolateral exposure, the TEER was significantly decreased compared to apical exposure [81].

Differential effects were observed in the decrease of the TEER of IPEC-1 cells after a 24-h exposure to 10 µM of DON or acetylated derivatives, and the toxins were ranked in the following order of toxicity: 15-ADON >> DON > 3-ADON [51]. Similar data were obtained using human Caco-2 cells, confirming the differential effects of toxins of a close chemical structure [84].

#### 5.2.2. Effects on Intestinal Permeability

The observed reduction in the TEER induced by trichothecenes can be due to an alteration of the tight junction barrier properties, but also to an effect on the plasma membrane, such as alterations in transcellular ion transport [85]. It is thus of interest to determine the effect of these toxins on the paracellular permeability of a tracer, such as lucifer yellow or dextran (Table 3).

Kasuga *et al*. [62] demonstrated a significant increase in the permeability of lucifer yellow in human Caco-2 and human T84 cells treated with DON. Similarly, we observed that DON increased the paracellular permeability of human Caco-2 cells and porcine IPEC-1 cells to 4 kDa dextran in a time and dose-dependent manner [71]. Akbari *et al*. [70] observed during 24 h that DON induced the dysfunction of the epithelial barrier of a Caco-2 cells by measuring the decline in impedance values. DON and its acetylated derivatives exhibit differential effects on 4 kDa dextran permeability of IPEC-1 cells, and after a 24 h exposure to 10 µM of toxin, they were ranked in the following order of toxicity: 15-ADON >> DON > 3-ADON [51].

This effect of DON on paracellular permeability was confirmed in rats chronically exposed to DON at 2 mg/kg of feed during 28 days. Pieces of jejunum mounted in an Ussing chamber showed a decrease of the TEER associated with an increase of permeability to 4 kDa dextran [50]. In pig explants mounted in Ussing chambers and exposed to DON *ex vivo*, we observed a two-fold increase in the paracellular passage of FITC-dextran across intestinal tissue treated with 20 µM and 50 µM of DON, when compared to untreated ones [71].

The numerous pores present in the basement membrane of the intestinal villi are essential for the communication of enterocytes with cells in the lamina propria. An 11-week exposure of pigs to DON at 2.2 to 2.9 mg/kg of feed led to an increase in the pore number in jejunum and potentially improved the antigen sampling in the intestinal epithelium [86].

#### 5.2.3. Effects on Bacterial Translocation

The impaired intestinal integrity could lead to the entry of luminal antigens and bacteria that are normally restricted to the gut lumen by the intestinal barrier function (Table 3). We observed that DON induces a dose-dependent translocation of a pathogenic strain of *Escherichia coli* across the porcine IPEC-1 epithelial cell monolayers [71]. An increased translocation of *Salmonella* *typhimurium* was observed in porcine IPEC-J2 exposed to low doses of DON, with undifferentiated cells being more sensitive than the differentiated ones [87]. Maresca *et al*. [72] demonstrated that among other mycotoxins, DON allowed the transepithelial passage of apically added non-invasive commensal bacteria across human Caco-2 cell monolayers. However, in this case, no modification of paracellular permeability evaluated by the TEER measurement or tracer flux was observed. Such an increase in the bacterial passage through intestinal epithelial cells after DON treatment could have major implications for human health in terms of sepsis and inflammation. In mice, DON-contaminated diet accelerates *S*. *enteritidis* infection [88] and transiently increases the severity of reovirus infection [89].

#### 5.2.4. Mode of Action

The mechanism underlying the trichothecene-induced impairment of the intestinal barrier function has been poorly investigated. The effect of DON on bacterial translocation could be related to the ability of this toxin to specifically decrease the expression of claudin proteins. Indeed, we have observed that, in porcine intestinal epithelial cell monolayers, the increased permeability was accompanied by a specific reduction in the expression of claudins. This increased permeability was also noted in pig explants treated with DON, and a reduction of claudin expression was described in the jejunum of piglets exposed to DON-contaminated feed [71]. This reduction of epithelial integrity through inhibition of the claudin-4 protein synthesis was observed in DON-exposed Caco-2 cells [73]. This decrease was not due to diminished transcription or increased degradation and was also observed for a tight junction-independent protein, *i.e.*, intestinal alkaline phosphatase [73]. As well as claudins, E-cadherin also plays a fundamental role in maintaining the epithelial architecture, and its expression is decreased in pig jejunal explants exposed to 10 µM of DON for 4 h [57]. The MAPK signaling pathway could be involved in the regulation of tight junction protein expression. Indeed, the ribotoxic stress induced by trichothecenes leads to the activation of members of the family of Src tyrosine kinases implicated as upstream regulators of a large number of intracellular signaling pathways [90]. They most likely represent critical signals that precede MAPK activation and the induction of resultant downstream responses [91]. In our study, we observed that the MAPK p44/42 ERK activation, induced by DON treatment, decreased the expression of claudin in correlation with a reduction in the barrier function of the intestine evaluated by TEER and paracellular permeability [92]. A recent study indicates that 6 h following the exposure of mice to 25 mg DON/kg bw, the distribution pattern of claudins 1, 2 and 3 was affected. In addition, the increase in the paracellular permeability, evaluated by the measure of FITC-dextran in the serum of mice, strengthened the hypothesis that the tight junction protein network is a target of DON [70]. We proposed a potential mechanism to explain the loss of the barrier function of intestinal epithelial cells following DON exposure, mediated by MAPK and claudin. The correlation between claudin 4 decreased expression and the MAPK activation was not observed in Caco-2 cells [73], suggesting a differential mechanism of action between porcine untransformed and human transformed intestinal epithelial cell lines.

Recently, we showed that the differential effects of DON and its acetylated derivatives on the intestinal barrier function were correlated, at the molecular level, with the exacerbated capacity of 15-ADON to activate MAPK ERK1/2, p38 and JNK, both in the intestinal cell line, explants and the jejunum from exposed animals, at a lower dose than DON and 3-ADON, and to decrease the expression of the tight junction proteins, claudin 3 and 4 [51].

DON cannot only interact with epithelial cells on the apical side during intestinal passage and absorption, but following absorption in the stomach and upper small intestine, detectable concentrations of DON can be found in blood serum, potentially exposing epithelial cells from their basolateral side. Diesing *et al*. [81] demonstrated a differential decrease in the tight junction protein, claudin 3, in the IPEC-J2 cell line, depending on the route of application, whereas the protein, ZO-1, was unaffected by the treatment. In addition, using a comparative global genomic approach, they showed that the apical and basolateral challenges to epithelial cell layers trigger different gene response profiles paralleled with a higher susceptibility towards basolateral challenge. The genes regulated were involved in metabolism, genetic or environment information processing and cellular processes [93].

The data summarized in this paragraph, obtained in different species (mouse, pig, human) and using different models (cell cultures, explants, *in vivo* experiments) confirm that one of the main target of DON in the intestine is the tight junction protein network. The modulation of claudin proteins by DON correlated with the increased intestinal permeability observed *in vitro*, *ex vivo* and *in vivo*. In terms of human or animal health, the consequences of the DON-induced increase in intestinal permeability still have to be determined.

## 6. Genotoxic Effects of DON and Other TCTB

The data on the genotoxic effects of trichothecenes are scarce, and these toxins are classified in Group 3 (inadequate evidence) by the International Agency on Cancer Research [94]. As far as the intestine is concerned, the genotoxic potential of NIV and FUS-X were evaluated *in vitro* on the human intestinal epithelial cell line, Caco-2. In differentiated post-confluent cells, a short exposure (3 h) to NIV or FUS-X did not cause any DNA damage, whereas DNA damage was observed after 24 h or 72 h [66]. In HT-29 cells exposed to DON, Bensassi *et al*. [68] demonstrated that the increase in DNA damage was induced in a time-dependent manner. Interestingly, in mice exposed orally and intraperitoneally to NIV (50% of the LD_50_), Tsuda *et al*. [95] observed that NIV was genotoxic in the gastrointestinal tract, with the colon mucosa being preferentially damaged.

## 7. DON and Other TCTB Modulate the Intestinal Immune Response

The intestinal immune response involves the coordinated action of both immune (dendritic cells, macrophages, lymphocytes) and non-immune cells, including epithelial cells. Monocytes, macrophages, dendritic cells, as well as T- and B-lymphocytes can be cellular targets of DON and other TCT. Low to moderate toxin concentrations upregulate the expression of cytokines, chemokines and genes, inducing an inflammatory response, both transcriptionally and post-transcriptionally [60]. Not only immune cells, but also intestinal epithelial cells produce cytokines, crucial for the recruitment and activation of the immune system, including TGF-α, IL-1, IL-10, IL-15 and IL-18 [96]. Other cytokines, such as IL-1-α or β, IL-6, IL-8, TNF-α, MCP-1, CCL20 and GM-CSF, are also expressed by normal epithelial cells and are markedly upregulated in response to microbial infections. Intestinal epithelial cells also drive the development of dendritic cells: they release thymic stromal lymphopoietin that inhibits IL-12 production by dendritic cells and TGF-β and retinoic acid involved in the development of tolerogenic dendritic cells. In addition, thymic stromal lymphopoietin favors the release of a proliferation-inducing ligand (APRIL) and the B-cell activation factor of the TNF family (BAFF) by intestinal epithelial cell-conditioned dendritic cells and supports IgA class switching directly in the lamina propria [97,98].

### 7.1. DON and Other TCTB Induce Intestinal Inflammation

#### 7.1.1. Modulation of the Cytokine Production in Intestinal Tissue by TCTB

The effect of DON or TCTB on cytokine production in intestinal tissue has been evaluated in different studies (Table 4). The increase in the expression of IL-1β, IL-8, MCP1 and IL-6 in pig intestinal loops exposed to *Salmonella* *typhimurium* was potentiated when DON was co-exposed with the bacteria [87]. The consequence of the DON intake could be an increase in the susceptibility to *Salmonella* *typhimurium*, with a subsequent potentiation of the inflammatory response in the gut.

**Table 4:** DON modulates cytokine production by intestinal epithelial cells.

Toxin	Species/model	Concentration and duration exposure	Cytokine modulation	References
***In vitro* approach: intestinal epithelial cell lines**				
**DON**	**Human intestine 407 and Caco-2 cell lines**	0–3.3 µM 12 h [94]	↗ IL-8	[72,80,99]
		0–10 µM, 12 h [64]		
		0–16.9 µM, 48 h [66]		
	**Porcine IPEC-J2 cell line**	0.5 µM, 48 h	↗ IL-1b, IL-6, IL-8,	[100]
		2 µM, 48 h	↘ IL-1a, MCP1	
			↗ IL-1a, IL-1b, IL-6, IL-8, TNFa, MCP1	
	**Human intestine 407 cell line**	24 h pre-exposure to LPS endotoxin	↘ IL-8	[101]
		1.7 µM, 12 h		
***Ex vivo* and *in vivo* approaches**				
**DON**	**Porcine jejunal explants (*ex vivo*)**	10 µM, 24 h	↗ IL-21, IL-22, IL-23	[102]
			↘ FoxP3, RALDH1	
	**Porcine intestinal loops (*in vivo*)**	0–3.3 µM, 6 h	↗ IL-1b, IL-8, MCP1, IL-6	[87]
	**Broiler chickens (*in vivo*)**	10 mg DON/kg, 35 d	↘ IL-1β, IFN-g, TGFBR1	[103]
			→ TNF-α, IL-8, NF-κβ,	

In porcine jejunal explants, 10 µM of DON led to an increase in the expression of IL-6, IL-23 and IL-1β, but did not affect the expression of TGF-β and strongly repressed FoxP3 and RALDH1. These data suggest that in this model, DON mainly drives the intestinal immune system towards a Th17 response elicited by the Th17 helper lymphocytes, recently described as important mediators of the mucosal immunity, the defense against extracellular pathogens and autoimmunity [102]. Besides these direct effects, DON also potentiates the effects of pro-inflammatory stimuli, such as TLR-4 ligands, lipopolysaccharide (LPS) and bacteria on immune cells [104,105,106]. Exposure to LPS is common and can occur through infections, via gastrointestinal translocation of gut microflora, due to inflammatory bowel diseases, or gut injury [107]. In mice, simultaneous exposure of subtoxic intravenous doses of LPS and dietary DON caused a sequential elevation of IL1-β overexpression and severe apoptotic depletion of lymphoid tissue [108,109]. However, such a mechanism needs to be demonstrated in intestinal lymphocytes during dietary exposure to LPS and TCT.

A recent study demonstrated a downregulation of the expression of IL-1β, IFN-g and transforming growth factor beta receptor I (TGFBR1) in broiler chickens fed for 35 days with DON at 10 mg/kg feed, but no changes in TNF-α, IL-8 and NF-κB in the jejunum of the animals [103]. 

#### 7.1.2. Modulation of the Cytokine Production in Intestinal Epithelial Cells by TCTB

DON provokes intestinal inflammation *in vivo* [110], which results from a direct effect on the production of pro-inflammatory cytokines, especially IL-8, by intestinal epithelial cells. IL-8 is an early marker of the inflammatory process and is a potent chemo-attractant for leukocytes and T-lymphocytes underlying gut epithelial cells. IL-8 also enhances cell proliferation and controls the repair processes during injury of the intestinal mucosa or cytotoxic stress [111]. Several studies have shown that DON stimulates the secretion of IL-8 in various human intestinal epithelial cell lines [72,80,99]. Indeed, after exposure of Caco-2 cells to DON, a dose-dependent increase in IL-8 secretion through an NF-kB activity mechanism was observed. This effect was amplified upon pro-inflammatory stimulation, showing that DON exposure could cause or exacerbate intestinal inflammation. Moreover, Maresca *et al*. [72] have shown that direct IL-8 secretion from differentiated Caco-2 cells in response to DON is dependent on the ribotoxic-associated activation of PKR, NF-kB and p38. By contrast, DON-induced IL-8 secretion in human embryonic epithelial intestine 407 cells (Int407) was dependent on the activation of MAPK ERK1/2, but not on the activation of p38. This difference is probably due to the maturation status of the cells: differentiated mature Caco-2 cells *vs.* undifferentiated Int407 cells [99]. Moreover, DON modulates the production of several pro-inflammatory cytokines following a 48-h treatment of IPEC-J2 cells. A 2-µM exposure upregulates IL-1α, IL-1β, IL-6, IL-8, TNFα and MCP1, whereas a 0.5-µM exposure upregulates IL-1β, IL-6, IL-8 and downregulates IL-1 and MCP1 [100]. The consequence of the production of pro-inflammatory cytokines is the modulation of the intestinal tight junction barrier, potentially favoring an increased translocation of luminal antigens [112].

The detection of bacteria by intestinal epithelial cells, which induces IL-8 secretion, is known to be mediated through the interaction of bacteria flagella with the cellular Toll-like receptor 5 [113]. An indirect pro-inflammatory effect of mycotoxins could result from an alteration of the intestinal barrier function, allowing the transepithelial passage of non-invasive commensal bacteria. Indeed, high doses of DON (around 100 µM) compromise tight junctions and allow the transepithelial passage of apically added non-invasive commensal bacteria [72]. In the same study, the authors showed that DON at 1 and 10 µM potentiates the effects of basolaterally added bacteria on the secretion of IL-8 by human intestinal epithelial cells.

The inflammation processes act to maintain tissue homeostasis, but as some cytokines are potent mediators of potentially damaging tissue responses, several mechanisms exist to ensure that the effects of these cytokines are restricted [114]. Recently, DON exposure was demonstrated to suppress BAFF gene expression via the induction of SOCS3 in human enterocytes. As TCT are ribosomal stress agents, their ingestion could exert adverse effects on the regulation of BAFF, a vital cytokine for B-cell development [115].

Moon *et al*. [101] showed that human epithelial cells are less responsive to DON-induced IL-8 production after pre-exposure to the endotoxin LPS. As the intestine of newborn infants becomes established with the normal microflora, their epithelium can recognize the external bacteria components. After further constitutive experience of the commensals and their endotoxins, the epithelium becomes hypo-responsive to the normal microflora and controls its associated physiological inflammation [116]. The mechanism of the hypo-production of IL-8 in intestinal epithelial cells is the extended production of the DON-induced proliferator-activated receptor γ (PPAR-γ) after pre-exposure to endotoxin. DON increased PPAR-γ gene expression, which was transiently maintained, but endotoxin pre-exposure extends the duration of DON-induced PPAR-γ expression, thus sensitizing cells to induce an extended PPAR-γ in response to DON treatment. Constitutively-expressed PPAR-γ on the intestinal epithelial surface may trigger the tolerance to the normal microflora and its associated inflammation [117]. For example, the impaired expression of PPAR-γ has been observed in inflammatory bowel diseases. The results demonstrated by Moon *et al*. [101] suggest that there is a potential risk of mucosal inflammation after DON exposure in young infants compared with endotoxin-tolerant adults.

### 7.2. DON and TCTB May Interfere with the Intestinal Homeostasis

As described previously, epithelial cells act as initiators, mediators and regulators in innate and adaptive immune responses, as well as in the transition from innate immunity to adaptive immunity. Dendritic cells collaborate as sentinels against foreign particulate antigens by building a transepithelial interacting cellular network. During inflammatory and immune responses, epithelial cells express pattern-recognition receptors to trigger a host defense response and interact with dendritic cells to regulate antigen sensitization and release cytokines to recruit effector cells [118]. Moreover, macrophages and T-cells are also able to modulate dendritic cell functions [119]. The effects of TCTB on the different cell types involved in gut homeostasis can explain various pathologies associated with the ingestion of mycotoxin contaminated food.

Firstly, DON can potentiate the effect of IL1-β on IL-8 secretion and increase the transepithelial passage of commensal bacteria [71,72]. IL-8 has been implicated in many chronic diseases, ranging from inflammatory bowel disease [120,121] to rheumatoid arthritis [122]. Then, in addition to potentially exacerbating established intestinal inflammation, this mycotoxin may thus participate in the induction of sepsis and intestinal inflammation *in vivo* [72]. Indeed, inflammatory bowel diseases, such as Crohn’s disease, are generally associated with the presence of adherent-invasive bacteria [123]. A hypothesis would be that at least in some cases, the ingestion of food contaminated with mycotoxins could be involved in inducing inflammatory bowel diseases [63,71,72]. 

The induction of proinflammatory cytokines, such as IL-6 by macrophages, plays a pivotal role, as they are directly linked to the differentiation of B-cells and to the stimulation of IgA secretion [124]. Prolonged feeding of DON causes a dramatic elevation in total serum IgA in mice. Moreover, dietary exposure to DON or NIV selectively upregulates membrane IgA-bearing cells in mouse Peyer’s patches [124]. IL-6 is critical to mucosal IgA immunity based both on its differentiative effects on IgA-committed B-cells and its production in the gut by macrophages and T-cells [125]. *In vivo* and *in vitro*, DON upregulates the IL-6 expression that drives the differentiation of IgA-committed B-cells to IgA secretion [126,127,128], mimicking the early stage of human IgA nephropathy. It will be interesting to study the implication of TCT on the release of soluble molecules, such as thymic stromal lymphopoietin (TSLP), retinoic acid and TNF-β by intestinal epithelial cells. Indeed, TSLP is shown to favor the release of BAFF and APRIL by conditioned dendritic cells and induces IgA switching directly in the lamina propria. The disruption of gut homeostasis induced by TCT could explain the modification of mucosal IgA responses, as well as the diverse immune-mediated pathology observed in response to these fungal toxins. 

## 8. Conclusions

The intestinal mucosa is the first biological barrier encountered by natural toxins, and consequently, it could be exposed to high amounts of dietary toxins. An increasing number of studies demonstrate that intestinal epithelial cells are targets for food contaminants, including mycotoxins [129,130,131]. 

In this review, we summarize the data concerning the ingestion of DON and other TCTB. These toxins induce intestinal pathologies in humans and animals, including necrosis of the intestinal epithelium. They also disturb the barrier function, potentially leading to the increased translocation of pathogens and an increased susceptibility to enteric infectious diseases. DON modulates the immune responsiveness of the intestinal mucosa, may interact in the cross-talk between epithelial cells and intestinal immune cells and could represent a predisposing factor to inflammatory diseases [102,132]. In farm and laboratory animals, dietary exposure to DON decreases growth performances. This is due to a local effect of the toxin altering the structure of the epithelium, reducing nutrient absorption by the enterocytes and modulating hormone production by enterochromaffin cells. A central effect of DON has also been described, involving the regulation of growth hormone production by inhibitors of cytokine signaling and the direct action of the toxin on the central neuronal network.

One important research field for the future will concern the impact of DON and other TCTB on the intestinal microbiota. Indeed, these toxins may directly target the microbiota [133]. In addition, the mucosal exposure to ribotoxic stress and the subsequent inflammatory responses may alter bacterial composition and, thus, reduce the microbial diversity. 

As mentioned in this review, the effects of DON and other TCTB have been investigated in different species, including man, laboratory animals, poultry and pigs. Among these species, pigs are of particular concern for at least two reasons: (i) due to the cereal-rich diet, pigs can be exposed to a high level of toxins; and (ii) the pig is one of the most sensitive species. In addition, because of the similarities in the intestinal tract, pigs can be considered as a good model for humans. In this species, several complementary approaches have been developed to investigate the effects of DON and other TCTB on the intestine. *In vivo* trials and cell culture models were used to study the long-term exposure to mycotoxins. Intestinal loops and explants enable multiple exposure conditions of the entire intestinal tissue to be investigated, but these models are limited to short-term exposure. The data obtained using these complementary approaches all show an impact of DON and other TCTB on the intestine, confirming the validity of the pig to investigate the effect of these toxins. Surprisingly, despite the available tool of this animal species, very few data have been obtained on the effect of DON on the intestine of rodents. This might be due to the fact that they are not very sensitive to this toxin.

The concentrations of toxins used in the *in vivo* trials presented in this review are, most of the time, in accordance with plausible levels of contamination. Similarly, in the different models of study of the intestine, cell culture or explants, the range of concentration used (generally 5–30 μM) is in accordance with these plausible levels [63]. Under these conditions of realistic exposure, TCTB produce deleterious effects on intestinal morphology and/or function. As highlighted recently by Maresca [41], the differences between the doses of DON affecting cell functions and the doses of DON susceptible to being present [134] in relation to the actual provisional maximum tolerable daily intake represent a low safety factor. Thus, DON can represent a risk to human health, mainly because of its effect on the intestinal and immune systems.

The data presented in this review focused on the effects of DON on the intestine. *Fusarium* species are able to synthesize, in addition to DON, other related toxins, among them the acetylated derivatives, the toxicity of which has been mainly investigated through *in vitro* experiments. Masked forms of mycotoxins, such as deoxynivalenol-3-β-d-glucoside (DON-3G), that result from the detoxification metabolism in plants, are an emerging problem [135]. The occurrence and toxicity of this new DON-metabolite are poorly documented, as well as its possible hydrolysis to the parent mycotoxin in the intestine. 

The toxicity of DON also needs to be addressed in the context of mycotoxin mixtures. Indeed, the co-occurrence of mycotoxins is likely to arise due to at least three different reasons: (i) most fungi are able to produce a number of mycotoxins simultaneously; (ii) food commodities can be contaminated by several fungi; and (iii) a complete diet is made up of various different food commodities [75]. Unfortunately, the toxicity of combinations of mycotoxins cannot always be predicted based upon their individual toxicities. Recent data suggest that the type of interaction depends not only on the type of toxin and their ratio, but also on the concentration of the toxin-mixture at a constant ratio [76]. More research is needed to understand the impact of mycotoxin combinations and to determine when synergistic interactions occur. These data are needed to assess the health risk due to the exposure of multi-mycotoxin contaminated food and feed [74].
